# Application of Pulse Compression Technique in High-Temperature Carbon Steel Forgings Crack Detection with Angled SV-Wave EMATs

**DOI:** 10.3390/s23052685

**Published:** 2023-03-01

**Authors:** Min He, Wenze Shi, Chao Lu, Guo Chen, Fasheng Qiu, Ying Zhu, Yuan Liu

**Affiliations:** 1 Key Laboratory of Non-Destructive Testing, Ministry of Education, Nanchang Hangkong University, Nanchang 330063, China; 2 State Key Laboratory of Acoustics, Chinese Academy of Sciences, Beijing 100190, China

**Keywords:** high-temperature carbon steel forgings, angled SV wave EMAT, Barker code pulse compression technique, circuit-field coupled FE model, signal-to-noise ratio

## Abstract

In order to solve the difficulty in localization and poor signal-to-noise ratio (SNR) of the angled shear vertical wave (SV wave) electromagnetic acoustic transducer (EMAT) in cracks detection of high-temperature carbon steel forgings, a finite element (FE) model of the angled SV wave EMAT detection process was established, and the influence of specimen temperature on the EMAT excitation, propagation, and reception processes was analyzed. A high-temperature resistant angled SV wave EMAT was designed to detect carbon steel from 20 °C to 500 °C, and the influence law of the angled SV wave at different temperatures was analyzed. Then a circuit-field coupled FE model of angled SV wave EMAT in the carbon steel detection process based on the Barker code pulse compression technique was established, and the effects of the Barker code element length, impedance matching method, and matching component parameters on the pulse compression effect were analyzed. In addition, the noise suppression effect and the SNR of the crack-reflected wave in the tone-burst excitation method and the Barker code pulse compression technique were compared. The results show that the amplitude of the block-corner reflected wave decreases from 556 mV to 195 mV, and the SNR decreases from 34.9 dB to 23.5 dB when the specimen temperature increases from 20 °C to 500 °C. When the temperature is 500 °C, the SNR of the crack-reflected wave obtained by the Barker code pulse compression technique can be improved by 9.2 dB compared to the tone-burst excitation method with 16 synchronous averages. The study can provide technical and theoretical guidance for online crack detection for high-temperature carbon steel forgings.

## 1. Introduction

High-temperature and high-pressure steam pipes under the action of high-temperature and high-pressure gas erosion, local corrosion thinning, severe oxidation, accompanied by creep, fatigue, and other deterioration forms cracks [1], which can easily cause steam leaks and even explosions and other serious accidents. In addition, carbon steel castings and forgings have surface and internal defects that cannot be avoided, due to factors such as equipment and process. In the high-temperature forging process, defects such as cracks, are prone to occur due to the temperature and the harsh environment of shaping [2]. Therefore, non-destructive testing and monitoring of high-temperature forgings are of great importance [3,4]. The electromagnetic acoustic transducer (EMAT) has the advantages of noncontact, no coupling agent, and high-temperature resistance, and it is suitable for harsh detection environments such as high-temperature, online, and rough surfaces [5,6,7]. But the EMAT also has some disadvantages, such as low conversion efficiency and poor signal-to-noise ratio (SNR) [8], which limit its wide application in industry. Therefore, it is necessary to improve the SNR of the ultrasonic wave by means of EMAT optimization design [9,10,11] and signal processing methods [12,13,14].

In terms of EMAT optimization design, Hill [15] used a periodic permanent magnet (PPM) combined with a racetrack coil to excite angled SH waves and investigated the relationship between excitation frequency and angle of the SH wave and width of the main lobe; Hill then verified that the number of PPM can suppress side lobe through experimentation. Ogi [16,17] investigated the relationship among wire spacing of meander-line coil (MLC), excitation frequency, and angle of the SV wave to give the optimal deflection angle range of angled SV wave and detected a crack with a depth of 0.05 mm using line-focusing SV wave EMAT with unequally spaced MLC. Nakamura [18] investigated the relationship between the focal position of point-focusing SV (PFSV) waves and the excitation frequency to further improve the amplitude and detection resolution of SV waves, and successfully detected a crack with a depth of 0.5 mm. Cai [19] proposed a modified one-side pitch-catch EMAT that improves the excitation of the horizontal magnetic field and contributes to the generation and reception of the Lamb wave, and Nakamura experimentally demonstrated that the measurement system improved by a factor of 3.3 over that using the conventional measurement system. Jia [20] proposed a novel multi-parameter optimization method to improve the conversion efficiency of PFSV-EMAT, which integrated the newly proposed hybrid surrogate modeling (HSM) method and the particle swarm optimization (PSO) algorithm for efficient optimization. The proposed HSM-PSO algorithm was used for the multi-parameter optimization of PFSV-EMAT, and the signal amplitude of the optimized PFSV-EMAT was improved by a factor of 12. Xie [21] proposed a new window function modulated EMAT design to generate unidirectional rayleigh waves to improve its defect detection capability. Thus, we can conclude that the EMAT optimal design is an effective way to enhance the SNR of the detected echoes, which can also be used in designing the high-temperature EMAT.

Regarding high-temperature EMAT detection technology, Kogia [6] established a PPM EMAT guided wave FE model for the problem of easily damaged solar absorber tubes and designed a water-cooling SH_0_ guided-wave EMAT probe to achieve online short-time high-temperature steel and stainless steel detection of 500 °C. Lunn [22] designed a water-cooling spiral coil EMAT for high-temperature inspection and performed thickness inspection on mild steel tubes and aluminum samples at 450 °C. Ogata S [23] used a spiral coil EMAT with a pulsed electromagnet, enabling carbon steel inspection at 600 °C without water cooling. Most of the research on high-temperature EMAT detection is focused on bulk waves or guided waves in high-temperature detection, and less research is conducted on high-temperature metal specimen crack detection. Ultrasonic signals with high SNRs cannot be obtained, due to disadvantages such as reduced EMAT conversion efficiency, significant ultrasonic attenuation coefficient, and strong electromagnetic interference in high-temperature environments [24,25]. The EMAT-optimized design has limited improvement in detection capability, the temperature significantly influences the amplitude and flight time of the ultrasonic signal, and it is difficult to acquire the ultrasonic signal accurately. In order to achieve non-destructive testing in high-temperature environments, scholars have adopted signal processing methods to reduce noise and improve the SNR of the ultrasonic signal. Huang [13] proposed an envelope modulation technique for noise removal; this algorithm could suppress small-amplitude and high-frequency noise mixed in the signal without damaging the main features of the signal, and it was experimentally verified with surface wave EMAT to eliminate noise and improve SNR. Sun [26] used the variational mode decomposition (VMD) method to decompose the received mixed-frequency signal, separating subsignals with different center frequencies and eliminating most noise. Then the continuous wavelet transform (CWT) method was used to perform time-frequency analysis on the decomposed subsignals to obtain the flight time of SH_0_ and SH_1_ mode guide waves. In addition, the authors applied the pulse compression technique to EMAT detection. HO K S [27] combined the pulse compression technique with wide-bandwidth EMAT to allow fast online measurement of metal plate thickness and high-resolution ultrasonic imaging. Izuka [28] designed a high-precision EMAT inspection system based on pulse compression with chirp signal excitation, which enabled online inspection of high-temperature continuous casting billets under maximum lift-off conditions of 5 mm. Cheng [29] designed three different encoded signals for high-temperature EMAT detection with pulse compression technology, and the SNR of the ultrasonic signal was able to be improved by 8.5 dB and the width of the ultrasonic signal was able to be reduced by 100% with the Barker code pulse compression technique compared to the conventional tone-burst excitation method.

However, when the pulse compression technique is applied to EMAT detection, the spectral characteristics of the EMAT detection system and the excitation signal bandwidth do not match, thus leading to spectral distortion and waveform distortion of the detected ultrasonic wave signal. At the same time, the material properties of metal change during high-temperature detection, which affects the frequency response characteristics of the detection system and leads to a decrease in the SNR and resolution of the detected signal after pulse compression. Therefore, this study combines an FE model of the angled SV wave EMAT detection process and an equivalent circuit model, and it considers the material properties of metals at different temperatures. The variation of angled SV wave with temperature is studied through the EMAT excitation process, the propagation process, and the reception process. By establishing a circuit-field coupled FE model of angled SV wave EMAT in carbon steel detection process with the Barker code pulse compression technique, the effect of code element length, impedance matching method, and impedance matching parameters of the Barker code on the pulse compression effect and the suppression effect of the Barker code pulse compression technique on external noise are studied. The experiments compared the SNR of the crack-reflected wave signal from the tone-burst excitation method with the Barker code pulse compression technique, and they verified the advantages of the Barker code pulse compression technique with angled SV wave EMAT in the detection of cracks in high-temperature carbon steel.

## 2. Circuit-Field Coupled FE Model of an Angled SV Wave EMAT in High-Temperature Carbon Steel Inspection

### 2.1. EMAT Excitation and Reception Equivalent Circuit Model

**Figure 1:**
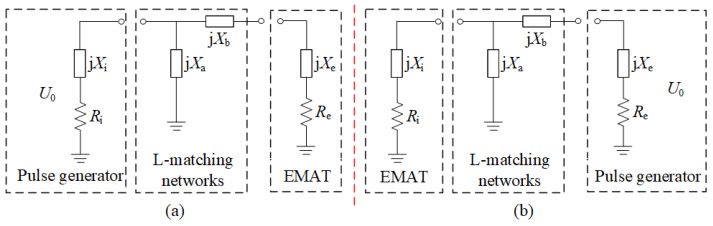
EMAT excitation equivalent circuit model (**a**) Impedance matching method I (*Q* > 0) Ⅱ (*Q* < 0) (**b**) Impedance matching method III (*Q* > 0) Ⅳ (*Q* < 0).

The EMAT excitation equivalent circuit model is shown in Figure 1, which mainly consists of a pulse power amplifier, LC impedance matching, and EMAT coil excitation equivalent impedance [30]. The pulse power amplifier can be equivalent to a voltage source *U*_0_ with internal resistances *R*_i_. The impedance matching network can be equated to a combination of capacitance or inductance with impedance values of j*X*_a_ and j*X*_b_, and the excitation of the EMAT coil can be equivalent to the resistance of *R*_e_ and the impedance of the j*X*_e_ inductance.

Four impedance matching methods—I, II, Ⅲ, Ⅳ—exist for the EMAT excitation equivalent circuit model [31]. Impedance matching methods I and II are shown in Figure 1a, and when the EMAT excitation equivalent circuit satisfies the conjugate matching condition, the impedance matching network parameters *X*_a_ and *X*_b_ can be obtained from Equation (1) [31] as follows.

(1) $$\left\{\begin{array}{l} X_{a}=-\frac{R_{i}{}^{2}+X_{i}{}^{2}}{X_{i}+QR_{i}}\\ X_{b}=QR_{e}-X_{e} \end{array}\right.$$

where the impedance matching quality factor *Q* [32] is defined by the equation:

(2) $$Q=\pm \sqrt{\frac{R_{i}}{R_{e}}\left[1+{\left(\frac{X_{i}}{R_{i}}\right)}^{2}\right]-1}$$

The input impedance *R*_i_ of the preamplifier in the EMAT reception circuit is 50 Ω, so when the coil impedance satisfies less than 50 Ω, the value *Q* exists. Impedance matching I and II correspond to *Q* values taken as “+” and “−”, respectively. As shown in Figure 1b, when switching the position of the RF power amplifier and the EMAT, the *Q* value is greater than 0 and lower than 0, corresponding to the impedance matching methods Ⅲ and Ⅳ, respectively.

**Figure 2:**
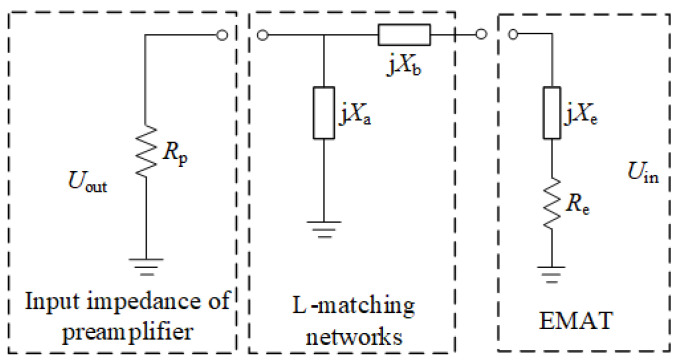
EMAT receiving equivalent circuit model.

The EMAT receiving equivalent circuit model is shown in Figure 2, which consists mainly of the input impedance of the preamplifier, the LC impedance matching circuit and the equivalent impedance of the receiving EMAT coil [32], which can be simplified to the impedance of *R*_e_ and *X*_e_. The LC impedance matching circuit consists of capacitors or inductors with impedance values j*X*_a_ and j*X*_b_.

### 2.2. FE Model of the Detection Process with Angled SV Wave EMAT

**Figure 3:**
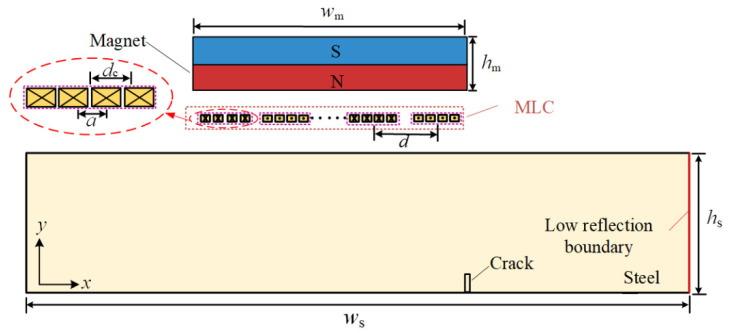
Schematic diagram for the design parameters of the angled SV wave EMAT.

Figure 3 shows a schematic diagram for the design parameters of the angled SV wave EMAT, and the conversion mechanism is described in the literature [16,17]. Since the biased magnetic field direction of the permanent magnet in the MLC EMAT is perpendicular to the specimen surface, and the magnetostrictive mechanism is small in proportion [33], only the Lorentz force mechanism is considered in this study.

Figure 3 is a schematic diagram of the angled SV wave EMAT, and its parameter values are shown in Table 1. The width *w*_s_ and height *h*_s_ of the carbon steel specimen are 160 mm and 50 mm, respectively. An MLC with 14 turns is taken, and each turn of the coil is composed of four copper wires, whose width *a* and spacing *d*_c_ are 0.15 mm and 0.3 mm, respectively. The turn spacing *d* is 1.5 mm. The magnet’s width *w*_m_ and height *h*_m_ are 25 mm and 30 mm, respectively. The excitation frequency of the tone-burst signal is 2 MHz. The center frequency of the code element of the 13-bit Barker code is 2 MHz, and the duration of the code element is 2 μs. A crack of 2 mm deep and 1 mm wide is prefabricated in the bottom border of the specimen. A 0.1 mm thick copper backplate lies underneath the magnet, which can avoid generating ultrasonic waves in the magnet and improve the SNR of ultrasonic waves.

**Figure 4:**
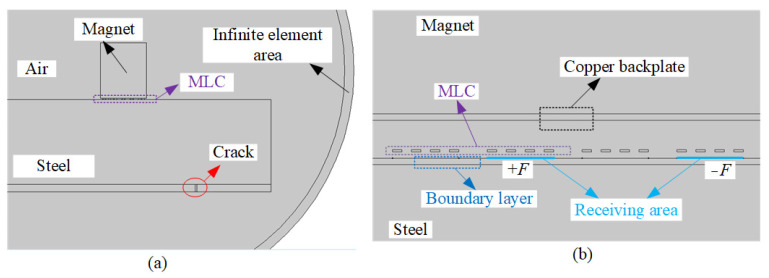
FE model of detection process with angled SV wave EMAT. (**a**) whole view (**b**) partial view.

The FE model of the angled SV wave EMAT detection process is shown in Figure 4. The permanent magnet, carbon steel specimen, and MLC are mapped with a mesh size of 0.5 mm, 0.15 mm, and 0.05 mm, respectively, and the air domain uses a free triangular mesh with a mesh size of 2 mm. A boundary layer grid is applied at the upper boundary of the specimen for the grid subdivision of the conversion area. Metal conductivity and elastic modulus corresponding to different temperatures are shown in Table 2 [34,35,36,37].

### 2.3. Circuit-Field Coupled Modeling Detection Process of Angled SV Wave EMAT

**Figure 5:**
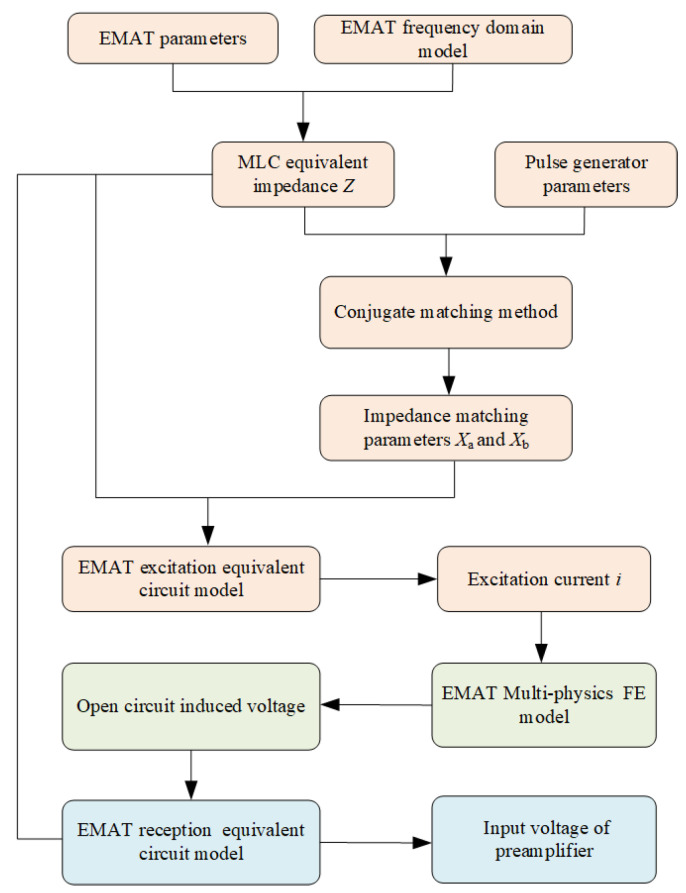
Circuit-field coupled FE modeling detection process of angled SV wave EMAT.

The circuit-field coupled FE model of the angled SV wave EMAT detection process based on the tone-burst excitation method consists of three main parts, as the flow chart shown in Figure 5 indicates.

Part I: The transient current of the excitation coil is solved based on the angled SV wave EMAT excitation equivalent circuit model. Based on the frequency domain model of the MLC EMAT impedance solution, the relevant parameters of the EMAT coil are set to obtain the equivalent impedance of the EMAT coil. The impedance matching parameters *X*_a_ and *X*_b_ are obtained by substituting the pulse generator parameters and the EMAT equivalent impedance parameters into the impedance matching model. The excitation current passing the EMAT coil is obtained by substituting the equivalent circuit model of excitation.

Part II: A multiphysics field FE model is based on the ultrasonic excitation, propagation, and reception processes of the angled SV wave EMAT to solve the open-circuit induced voltage of the EMAT. The input of the model is the excitation current calculated in Part I, and the output is the open-circuit induced voltage.

Part III: The impedance matching parameters and coil equivalent impedance obtained from Part I and the open-circuit induced voltage from Part II are substituted into the EMAT receiving equivalent circuit model to obtain the input voltage of the preamplifier.

## 3. Analysis of the Influence Factor of the Ultrasonic Echoes from the Angled SV Wave EMAT in the Detection of High-Temperature Carbon Steel

### 3.1. Influence of Temperature on the Excitation Ultrasonic Wave of Angled SV Wave EMAT

**Figure 6:**
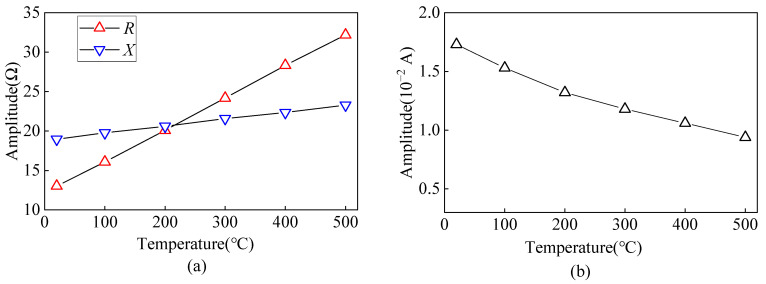
Variation curve of (**a**) equivalent impedance and (**b**) excitation current from the angled SV wave EMAT coil at different temperatures.

The equivalent impedance of the EMAT coil at different temperatures is calculated by the FE model in the frequency domain with a frequency of 2 MHz. Considering the effect of temperature on the conductivity of carbon steel, copper wire, and copper backplate, the equivalent impedance of the EMAT coil at different temperatures is obtained, as shown in Figure 6a. From Figure 6a, it can be seen that *R* and *X* of the EMAT coil increase with increasing temperature. When the temperature increases from 20 °C to 500 °C, the *R* and *X* of the EMAT coil increase by 59.57% and 18.58%, respectively, and the temperature has a more significant effect on the resistance *R* of the EMAT coil.

The input impedance *R*_i_ of the preamplifier in the EMAT reception circuit is 50 Ω. A set of j*X*_a_ and j*X*_b_ in the LC impedance matching circuit corresponds to a capacitance of 2.68 nF and an inductance of 0.237 μH, respectively. The curve of excitation current variation with different temperatures is shown in Figure 6b, and the amplitude of the excitation current decreases by 45.66% when the temperature increases from 20 °C to 500 °C.

**Figure 7:**
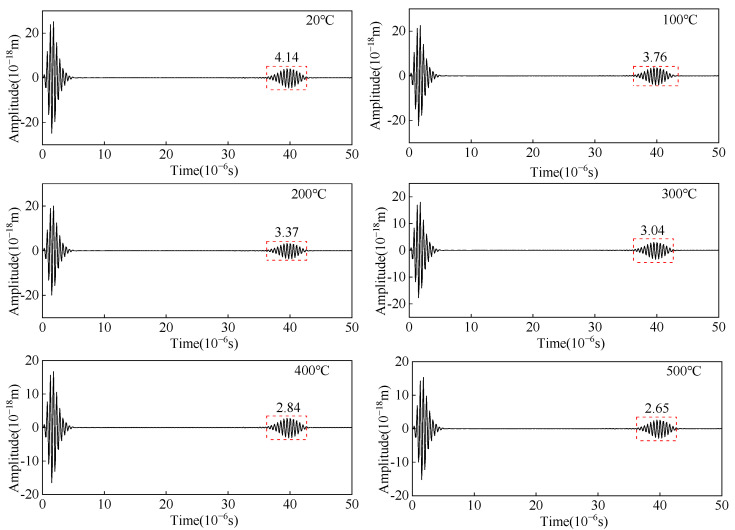
Excitation ultrasonic wave signal from the angled SV wave EMAT at different temperatures.

As shown in Figure 4, an odd turn line segment of equal length to the single turn coil is taken as the receiving area on the surface of the carbon steel specimen, and the line integral of the *x*-direction ultrasonic displacement component is calculated along the receiving area to obtain the excitation ultrasonic wave signal at different temperatures as shown in Figure 7. When the temperature increases from 20 °C to 500 °C, the amplitude of the excitation ultrasonic wave signal decreases by 35.99%. The conductivity of the copper wire and copper backplate decreases as the temperature increases, increasing the equivalent impedance of the EMAT coil, which results in a decrease in the excitation current obtained and thus a reduction in the amplitude of the EMAT excitation ultrasonic wave.

### 3.2. Influence of Temperature on the Propagation Process of Angled SV Wave

**Figure 8:**
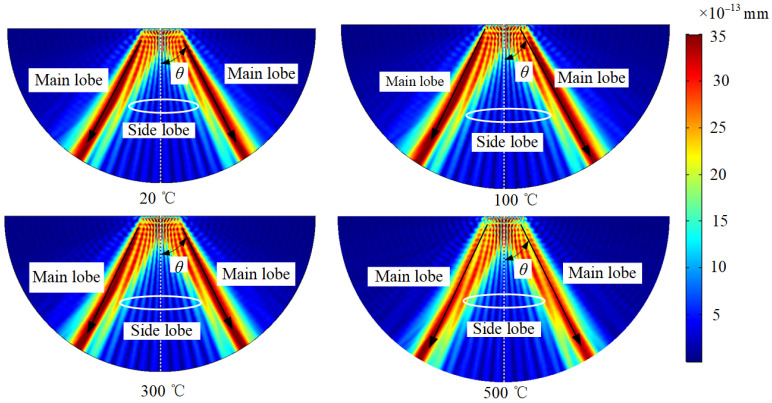
Acoustic radiation field of angled SV wave EMAT in carbon steel specimens at different temperatures.

**Figure 9:**
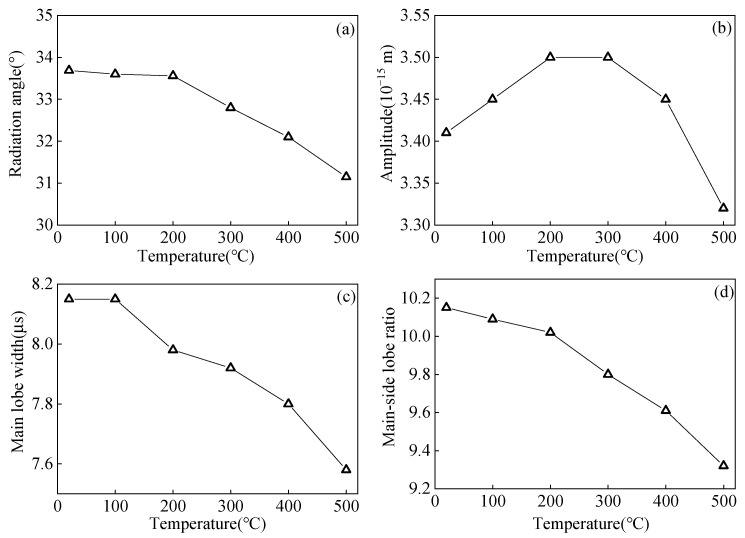
Influence of temperature on (**a**) radiation angle (**b**) main lobe amplitude (**c**) main lobe width (**d**) main-side lobe ratio of the angled SV wave.

The elastic modulus affects the acoustic radiation field and propagation velocity of the ultrasonic waves, so changes in the elastic modulus at different temperatures are considered to study the variation in the acoustic radiation field and propagation process of angled SV waves at different temperatures. The acoustic radiation field at different temperatures is shown in Figure 8, and Figure 9 is a variation graph of angled SV wave acoustic radiation field characteristics with temperatures. Among them, the radiation angle is the angle *θ* between the main beam and the vertical direction. The amplitude of the main lobe is the maximum component of the ultrasonic displacement component. The main-side lobe ratio is the ratio between the main lobe amplitude and the side lobe amplitude. The width of the main lobe is calculated using the −6 dB method. As the temperature increases, the radiation angle of the angled SV wave becomes smaller, and the main lobe amplitude first increases and then decreases, with a small overall trend. When the temperature increases, the SV wave beam will deviate from the crack position, reducing the SV amplitude. The higher the temperature, the more deviation arises. Since the main lobe width decreases as the temperature increases, the energy of the radiation field will gather, and the amplitude will increase. Moreover, the temperature affects the beam shape of the SV wave and modifies the focal position of the SV wave, which will respond to the changing trend of the main lobe amplitude. Thus, the main lobe amplitude increases from 20 °C to 200 °C and gradually decreases from 200 °C to 500 °C. The width of the main lobe decreases, and the main lobe width at 500 °C decreases by 0.57 μs compared to 20 °C. The main-side lobe ratio decreases with increasing temperature, and the main-side lobe ratio at 500 °C decreases by 8.17% compared to 20 °C.

**Figure 10:**
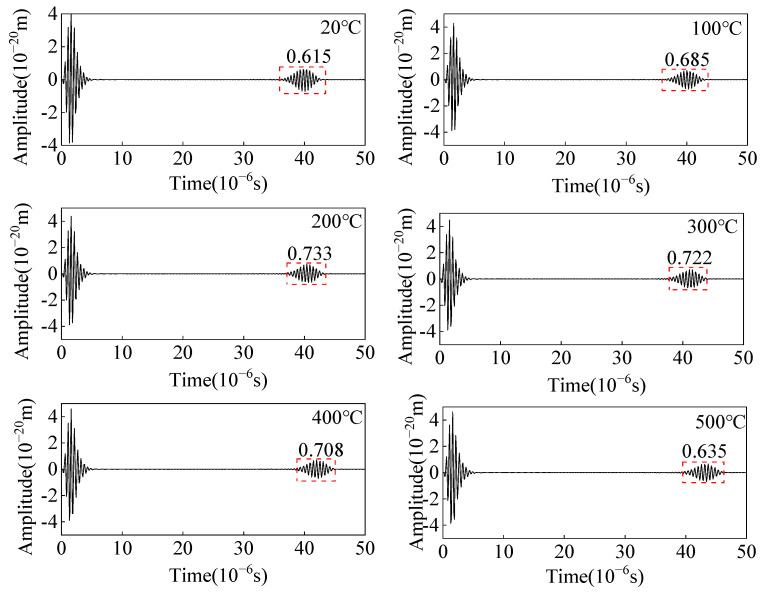
Ultrasonic displacement signal from angled SV wave EMAT in carbon steel specimens at different temperatures.

Changing the elastic modulus of the carbon steel specimen to study the amplitude and flight-of-time of the angled SV wave with temperature, and the ultrasonic wave signal at different temperatures is shown in Figure 10. As the temperature increases, the flight-of-time of the crack-reflected echo increases 3.3 μs from 20 °C to 500 °C. When the temperature increases from 20 °C to 200 °C, the amplitude of the crack-reflected echo increases and gradually decreases with increasing temperature from 200 °C to 500 °C. The main lobe amplitude increases from 20 °C to 200 °C, and gradually decreases with increasing temperature from 200 °C to 500 °C. When the defect is detected using the angled SV wave, the amplitude of the crack-reflected wave obtained shows the same trend as the main lobe amplitude.

### 3.3. Influence of Temperature on the EMAT Reception Process of Angled SV Waves

**Figure 11:**
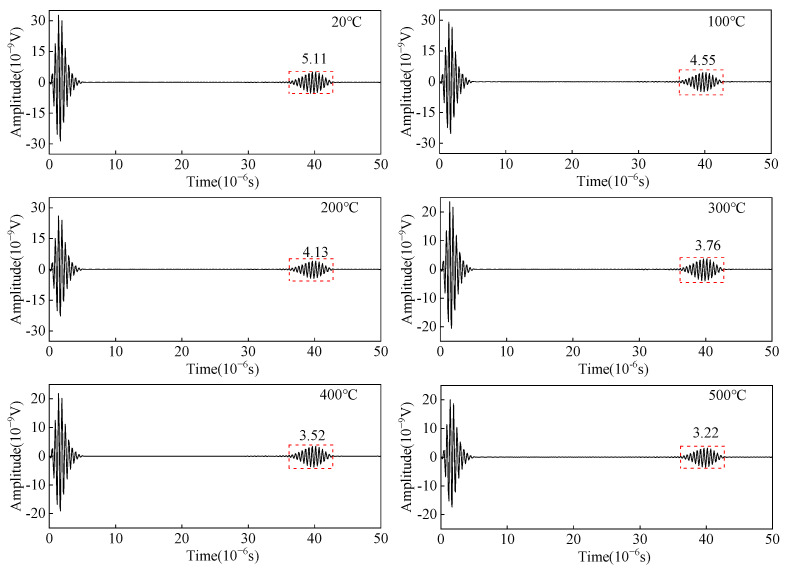
Open-circuit inducted voltage from angled SV wave EMAT in carbon steel specimens at different temperatures.

**Figure 12:**
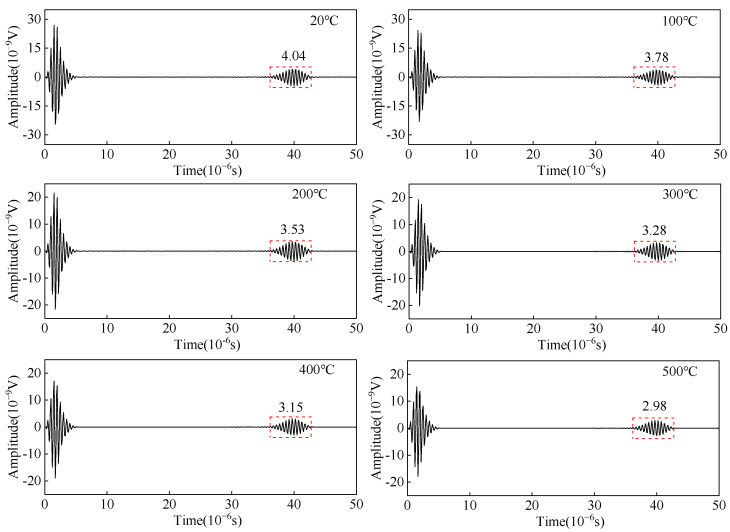
Input voltage of the preamplifier from the angled SV wave EMAT reception circuit with different temperatures.

As shown in Figure 4, a linear load *F* is applied on positive and negative phases on the surface of the carbon steel specimen with an equal-length segment of the single-turn coil. The change in the copper backplate conductivity, the coil conductivity, and the carbon steel specimen conductivity are considered to study the effect of temperature on the wave amplitude of the receiving angled SV wave EMAT. The open-circuit induced voltage of the receiving EMAT coil as the received ultrasonic signal is shown in Figure 11. From Figure 11, the amplitude of the crack-reflected wave decreases by 36.98% when the temperature increases from 20 °C to 500 °C. When we study the influence of the EMAT reception process of angled SV wave, only conductivity is considered without discussing the modulus of elasticity of carbon steel. As the temperature increases, the conductivity decreases. Thus, the skin depth is increased, resulting in a decrease in the eddy current density, and therefore the open-circuit inducted voltage drops. The open-circuit induced voltage is used as the voltage source of the receiving equivalent circuit, and the input resistance of the preamplifier is set to 50 Ω. With the existence of the impedance-matching network, the input voltage of the preamplifier is obtained at different temperatures, as shown in Figure 12. The input voltage of the preamplifier decreases by 26.23% when the temperature increases from 20 °C to 500 °C. When the open-circuit induced voltage is then passed into the equivalent reception circuit, the impedance of the equivalent receiving circuit increases with increasing temperatures, and the input voltage of the preamplifier becomes smaller.

### 3.4. Influence of Temperature on the Detected Ultrasonic Wave from Angled SV Wave EMAT

**Figure 13:**
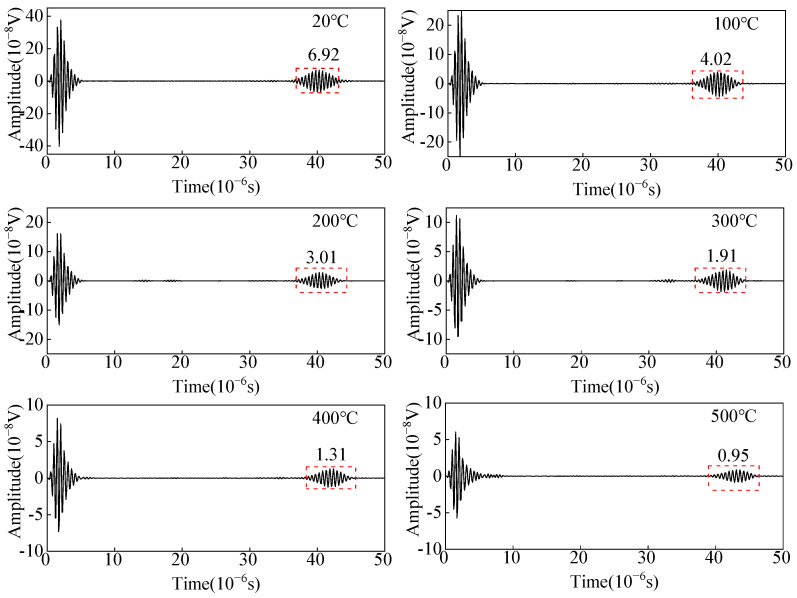
Detected ultrasonic wave signal obtained by the circuit-field coupled FE model of the angled SV waves EMAT.

To study the variation of a crack-reflected wave of angled SV wave EMAT in carbon steel specimens at different temperatures, the effects of temperature on coil conductivity, copper backplate conductivity, carbon steel conductivity, and elastic modulus are considered. The calculated equivalent impedance and impedance matching parameters of the MLC are substituted into the excitation equivalent circuit model to obtain the excitation current. The calculated excitation current is then substituted into the multiphysics FE model of the angled SV wave EMAT excitation process to obtain the open-circuit induced voltage of the receiving EMAT. The input voltage of the preamplifier is calculated by using the open-circuit inducted voltage obtained from the receiving EMAT, which is regarded as the voltage source in the receiver equivalent circuit, as shown in Figure 13. When the temperature increases from 20 °C to 500 °C, the input voltage of the preamplifier decreases by 86.27%. The input voltage of the preamplifier can accurately represent the received ultrasonic wave signal in practical ultrasonic wave detection. When the temperature increases, the conductivity of the copper wire and copper backplate decreases, the EMAT coil equivalent impedance becomes larger, and the excitation current decreases. Thus, the input voltage of the preamplifier drops. So, the amplitude of the detected ultrasonic wave signal decreases.

### 3.5. Experimental Verification of Angled Wave EMAT in High-Temperature Carbon Steel Inspection

**Figure 14:**
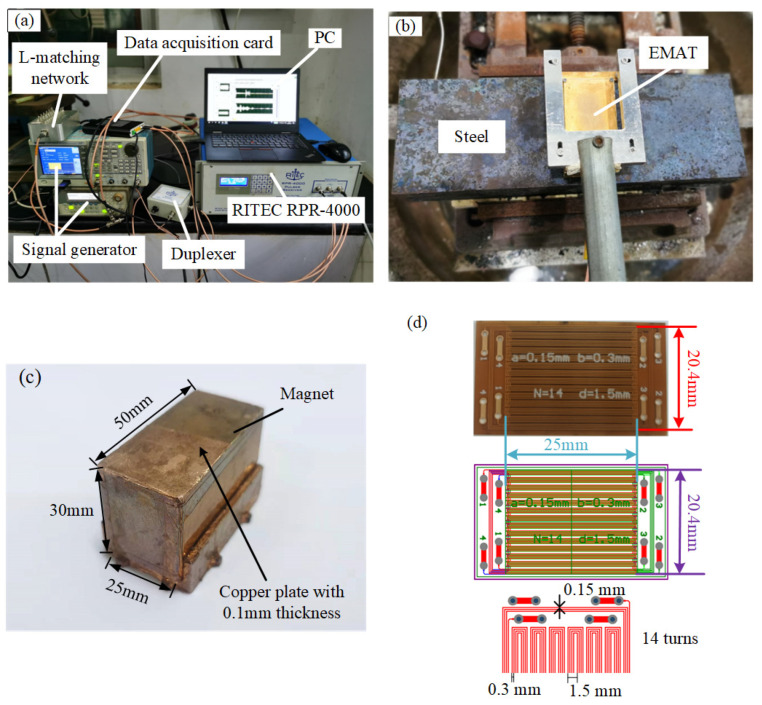
(**a**) EMAT experimental system with high-temperature steel, (**b**) Angled SV wave EMAT, (**c**) Magnet, (**d**) MLC.

Figure 14a shows the angled SV wave EMAT detection system in high-temperature carbon steel. The signal generator generates the tone-burst excitation signal and the Barker code excitation signal, and the RITEC RPR-4000 amplifies them to obtain high-frequency and high-amplitude excitation currents. Impedance matching is used to achieve a conjugate match between the equivalent impedance of the power amplifier and the EMAT coil. The receiving ultrasonic signal is filtered and amplified internally and transmitted to the PC by the data acquisition card. Then the display and storage of the ultrasonic signals are realized through the LABVIEW software interface. The angled SV wave EMAT with a duplexer can be used to achieve angled SV wave reflection detection.

Figure 14b shows a physical drawing of the angled SV wave EMAT in a carbon steel specimen with sizes of 300 mm long, 100 mm wide, and 50 mm high. The EMAT consists of a permanent magnet and an MLC, where the permanent magnet has dimensions of 50 mm long, 25 mm wide, and 30 mm high, as shown in Figure 14c, and a copper backplate with 0.1 mm thickness is used to wrap the permanent magnet. The MLC of the angled SV wave EMAT is printed on a flexible printed circuit board (FPCB), as shown in Figure 14d. The settings for the experimental verification are the same as those for the FE simulation.

**Figure 15:**
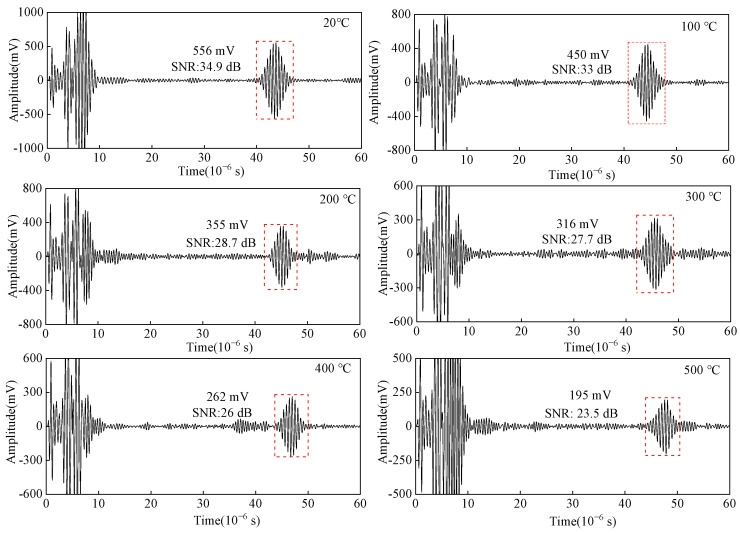
Detected reflected ultrasonic wave signal of angled SV wave EMAT in carbon steel specimens from 20 °C to 500 °C.

**Figure 16:**
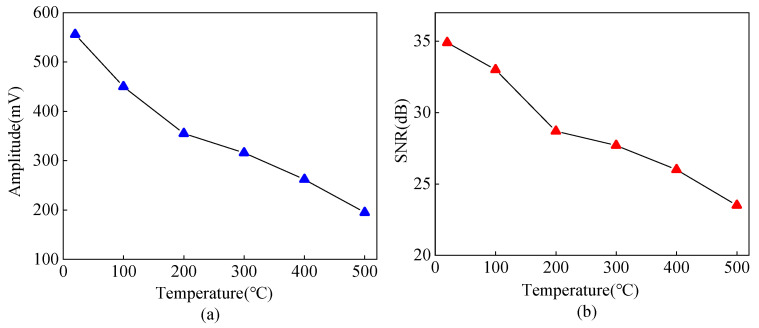
(**a**) Amplitude (**b**) SNR of the block-corner reflected wave from angled SV wave EMAT in carbon steel specimens from 20 °C to 500 °C.

When using the tone-burst excitation method with 2 MHz, the excitation cycle is five, and the synchronous average is 16. The carbon steel of 20–500 °C is detected by the pulse-reflective method, and the block-corner reflected wave of the carbon steel specimen is shown in Figure 15. The curves of amplitude and SNR from angled SV wave EMAT in the carbon steel specimen detection with temperatures are shown in Figure 16. When the temperature increases from 20 °C to 500 °C, the amplitude of the block-corner reflected wave decreases by 64.9%, and its SNR decreases by 11.4 dB. As the temperature increases, the equivalent impedance of the MLC increases, the excitation current decreases, the input voltage of the preamplifier of the receiving circuit decreases, and the ultrasonic attenuation coefficient also increases with the growing temperature. Thus, the amplitude of the ultrasonic wave signal decreases, which is consistent with the conclusion obtained from the simulation in Figure 13.

## 4. Application of Pulse Compression Technique in High-Temperature Angled SV Wave EMAT Carbon Steel Detection

### 4.1. Implementation Process of Barker Code Pulse Compression Technique and Noise Suppression Capability

**Figure 17:**
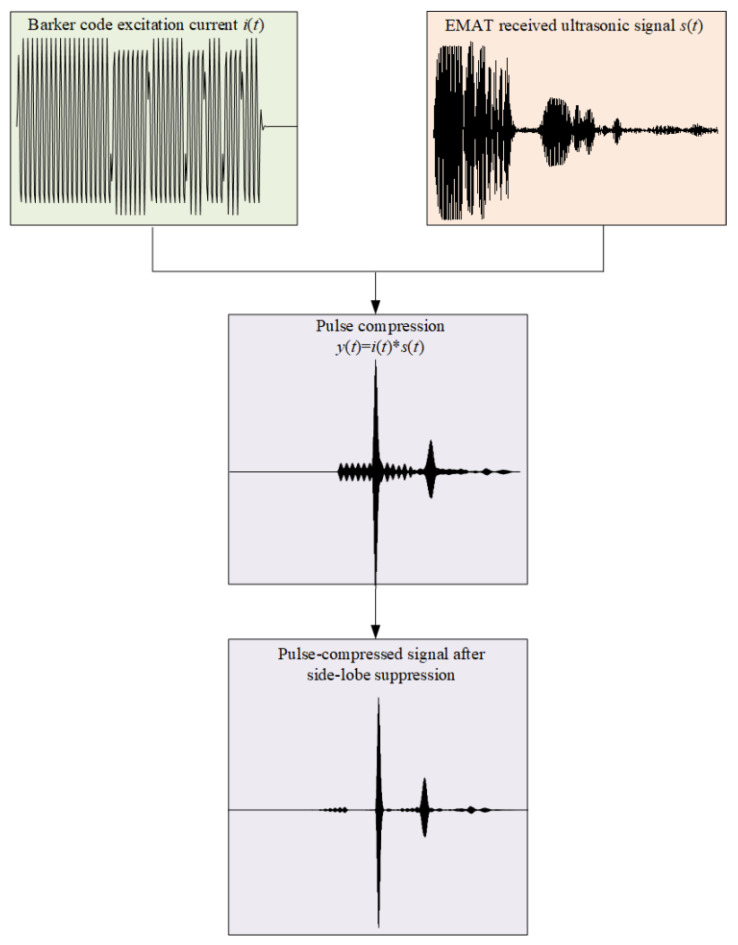
Barker code pulse compression technique implementation process.

The Barker code is a kind of nonperiodic binary code set with special laws, whose code elements only take +1 or −1. The signal has good autocorrelation characteristics and noise suppression characteristics. The pulse compression process is shown in Figure 17. A sinusoidal signal is used as the code element of the Barker code signal, and then the Barker code signal is loaded into the EMAT as the excitation current signal *i*(*t*) of the EMAT. After the calculation of the multiphysics field FE model, the EMAT received signal *s*(*t*) can be obtained. The excitation current signal *i*(*t*) and the received ultrasonic *s*(*t*) are convolved to realize the pulse compression process, and the final pulse-compressed signal is obtained after side-lobe suppression.

**Figure 18:**
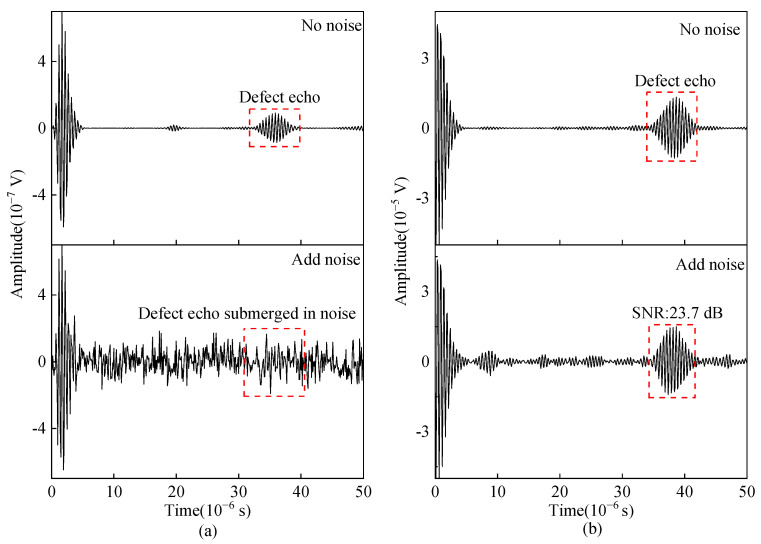
Simulated ultrasonic signal from angled SV wave EMAT with (**a**) the tone-burst excitation method (**b**) the Barker code pulse compression technique.

**Figure 19:**
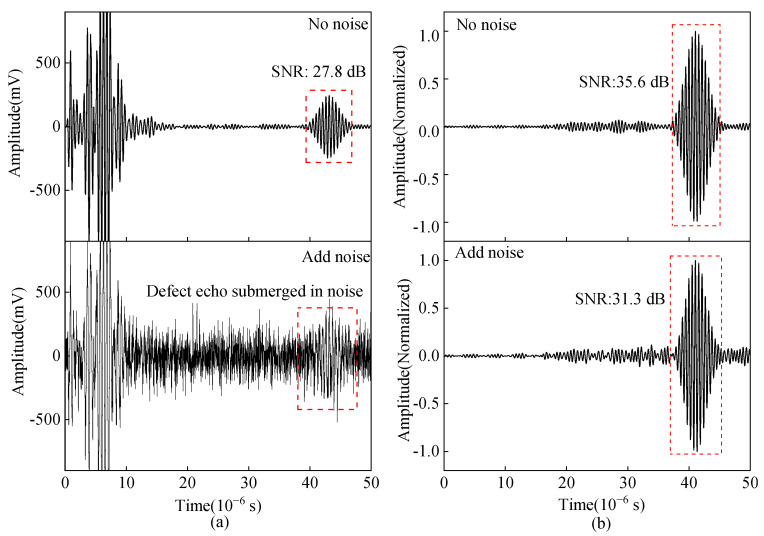
Experimental ultrasonic signal from angled SV wave EMAT with (**a**) tone-burst excitation method (**b**) Barker code pulse compression technique.

In the actual pulse power amplifier, the output signal has a sine or cosine signal different from the excitation frequency, which is usually the power amplifier’s own noise and will affect the SNR of the detected signal from EMAT. Therefore, random noise is added to simulate the power amplifier’s own output noise and external ambient electromagnetic noise. A tone-burst signal with a frequency of 2 MHz and a 13-bit Barker code signal with a code element duration of 2 μs were selected for simulation and experimental verification of the noise suppression capability. As shown in Figure 18, the ultrasonic signal obtained from the tone-burst excitation method is submerged in noise, while the SNR of the ultrasonic signal obtained by the pulse compression technique can still reach 23.7 dB, which can clearly identify defect waves. Figure 19 shows the contrast of the experimental signal with the tone-burst excitation method and the Barker code pulse compression technique after adding noise. It proves that the Barker code pulse compression technique is beneficial for suppressing external noise interference and improving the SNR of the reflected ultrasonic wave signal.

### 4.2. Circuit-Field Coupled Analysis of Angled SV Wave EMAT

**Figure 20:**
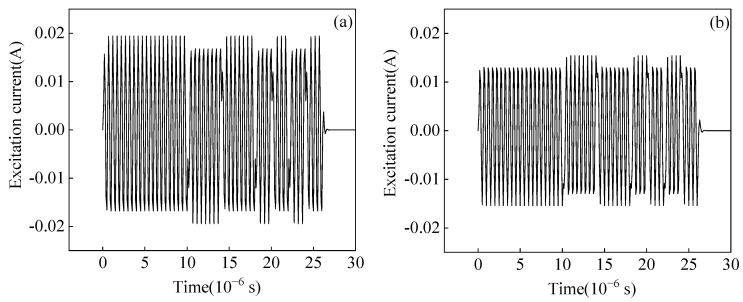
Excitation current calculated by EMAT excitation equivalent circuit with (**a**) Impedance matching method I (**b**) Impedance matching method II.

The impedance matching method and parameters will affect the pulse compression effect. Only two impedance matching methods I and II exist through the calculation, and the calculated excitation current is shown in Figure 20. The calculated excitation currents vary greatly using the same MLC and different impedance matching. This is due to the inherent frequency response characteristics of the EMAT probe and the different impedance-matching methods in the circuit.

**Figure 21:**
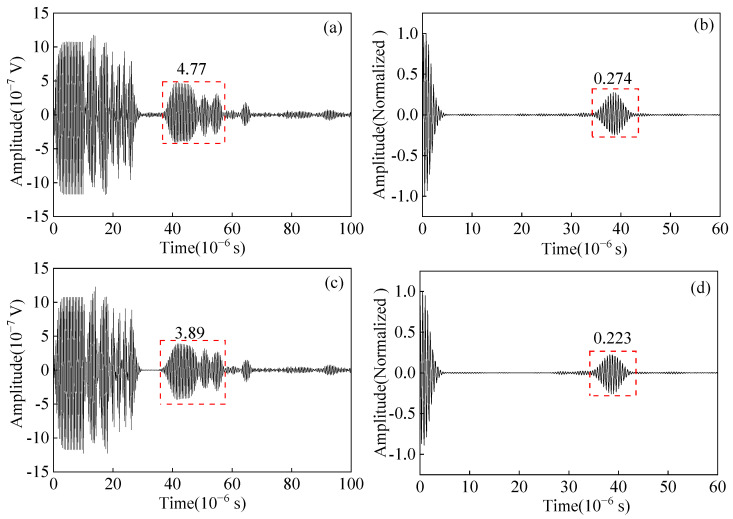
(**a**) Signal before pulse compression (**b**) pulse compressed signal with impedance matching method I; (**c**) signal before pulse compression (**d**) pulse compressed signal with impedance matching method II.

The input voltages of the preamplifiers obtained using impedance matching methods I and II are shown in Figure 21a,c. The spectral variability of the excitation currents results in different amplitudes of the input voltages of the received preamplifiers. The input voltage amplitudes of the preamplifier corresponding to the impedance matching method I are greater than that of impedance matching method II. After pulse compression processing, the pulse-compressed signal is obtained, as shown in Figure 21b,d. The amplitude of the main lobe corresponding to impedance matching mode I is 22.8% higher than that of impedance matching mode II.

**Figure 22:**
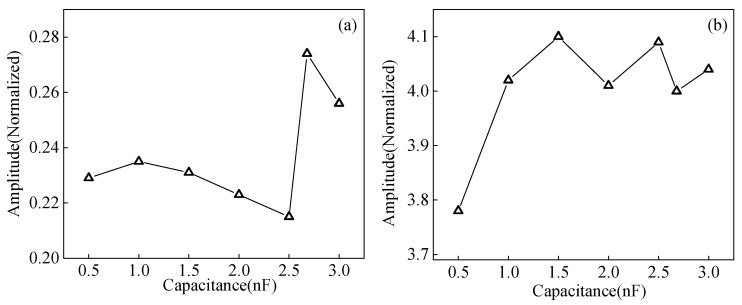
Influence of impedance matching capacitance on (**a**) the amplitude of the main lobe (**b**) the width of main lobe of the pulse-compressed signal in carbon steel specimen.

**Figure 23:**
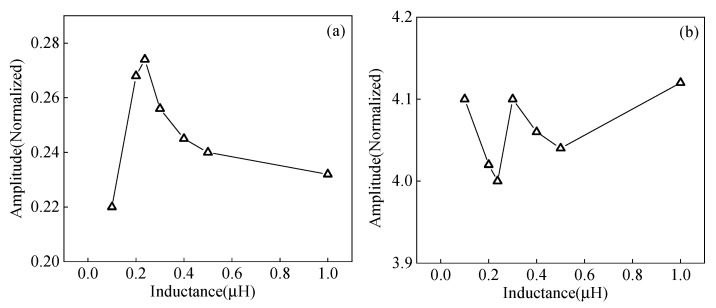
Influence of impedance matching inductance on the (**a**) amplitude (**b**) width of the main lobe of the pulse compressed signal in a carbon steel specimen.

I-impedance matching mode is selected to study the effect of impedance matching parameters on the pulse compression effect. When the matching inductance is the perfect impedance matching value of 0.237 uH, the EMAT probe parameters and matching inductance are kept unchanged, and only the capacitance value is changed. The curves of the main lobe amplitude and main lobe width of the pulse compressed signal with capacitance are obtained and shown in Figure 22. As the matching capacitance increases, the main lobe amplitude of the pulse-compressed signal tends to decrease, then increase, and then decrease. The main lobe amplitude of the pulse compressed signal reaches the maximum value when the matched capacitance is the optimal parameter. The overall effect of the change in capacitance on the width of the main lobe is small. When the matching capacitance is in the perfect impedance matching value of 2.68 nF, the EMAT parameters and matching capacitance remain unchanged. Only the matching inductance value needs to be changed to get the variation curve of the main lobe amplitude and main lobe width of the pulse-compressed signal, as shown in Figure 23. As the matching inductance increases, the main lobe amplitude of the pulse-compressed signal shows a trend of first increasing, then decreasing, and the main lobe amplitude of the pulse-compressed signal is the largest when the matching inductance is the optimal parameter. The overall effect of the change in inductance on the width of the main lobe is small. When the pulse compression technique is applied to the EMAT, the changes in matching capacitance and inductance will affect the frequency response characteristics of the detection system, causing changes in the detected echo. Thus, the effect of pulse compression will change, resulting in changes in the amplitude and width of the echo after pulse compression. The impedance matching network can realize the conjugate matching between the equivalent impedance of the power amplifier and EMAT coil so that the EMAT efficiency can be optimized. When the impedance matching is the best matching value, the maximum amplitude of the ultrasonic signal is obtained. Therefore, the impedance matching parameters need to be adjusted to the best impedance matching during the experimental testing, and the pulse compression effect will be the best.

### 4.3. Analysis of the Effect of Code Element Length on Pulse Compression

**Figure 24:**
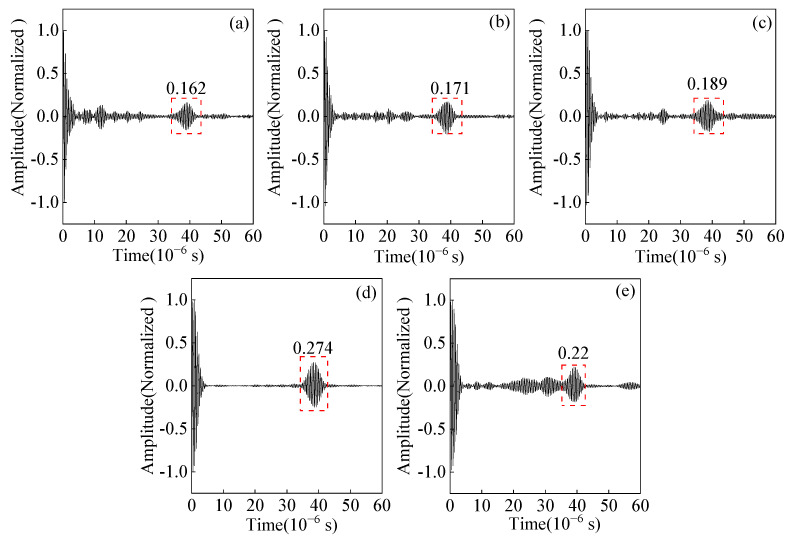
Effect of code element length on the main lobe amplitude of simulation signal from angled SV wave EMAT after pulse compression (**a**) T = 0.5 μs (**b**) T = 1 μs (**c**) T = 1.5 μs (**d**) T = 2 μs (**e**) T = 2.5 μs.

The length of the code element is an essential parameter of the Barker code pulse compression technique. A field-circuit coupled FE model is established to study the effects of different code element lengths of Barker code pulse compression. The ultrasonic signal obtained after the Barker code pulse compression with different code element lengths from 0.5 μs to 2.5 μs is shown in Figure 24. As the length of the code element increases, the amplitude of the main lobe first increases and then decreases, and the amplitude of the main lobe reaches the maximum value when the length of the code element is 2 μs. Continuing to increase the code element length to 2.5 μs, the main lobe amplitude decreases, and an obvious side lobe appears.

**Figure 25:**
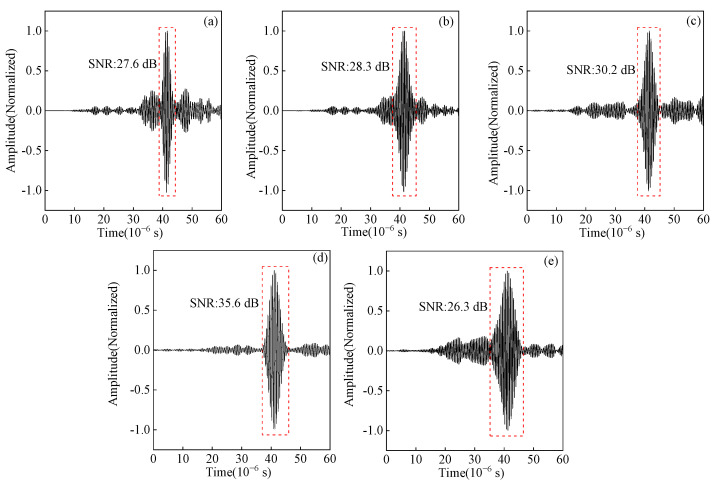
Effect of code element length on the main lobe amplitude of the experimental signal from angled SV wave EMAT after pulse compression (**a**) T = 0.5 μs (**b**) T = 1 μs (**c**) T = 1.5 μs (**d**) T = 2 μs (**e**) T = 2.5 μs.

**Figure 26:**
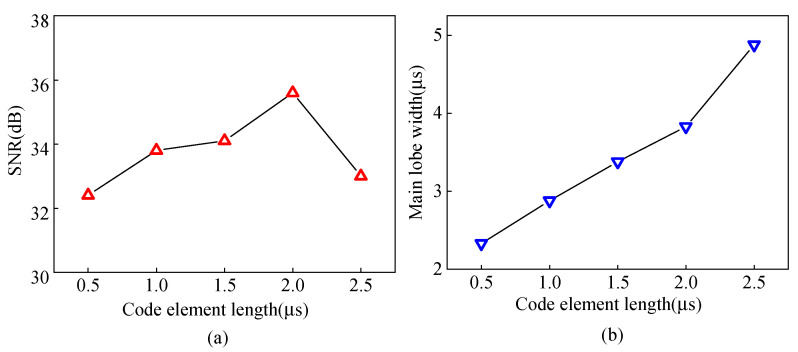
Curves of (**a**) SNR (**b**) main lobe width of defected waves from angled SV wave EMAT after pulse compression with different code length.

The experimental pulse compression signals corresponding to different lengths of code element are shown in Figure 25. Moreover, the variation curves of the SNR and wave packet width for different code element lengths are shown in Figure 26. When the code element length increases from 0.5 μs to 2 μs, the SNR of the defected echo increases by 3.2 dB, and the width of the wave packet increases by 1.5 μs. Increasing the code element length to 2.5 μs, the SNR decreases, the wave packet width increases by 1.05 μs compared to 2 μs, and the spatial resolution decreases. Appropriately increasing the code element length is beneficial for increasing the SNR of the ultrasonic signal, but the ability to improve the SNR is limited, and the increasing wave packet width will affect the spatial resolution. Therefore, a code element length of 2 μs is chosen as the optimal parameter.

### 4.4. Analysis of Angled SV Wave EMAT in High-Temperature Carbon Steel Crack Detection

**Figure 27:**
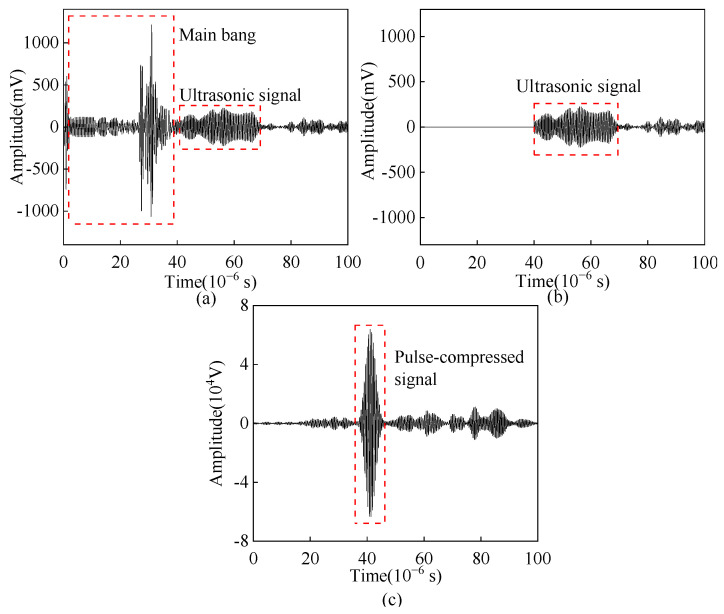
(**a**) Original A-scan signal (**b**) A-scan signal after removal of electromagnetic crosstalk (**c**) Pulse-compressed signal received by angled SV wave EMAT.

Figure 27a shows the original ultrasonic A-scan signal obtained from the angled SV wave EMAT experiment. It can be seen that the wave packet width of the original ultrasonic A-scan signal is large. To avoid the disturbance of electromagnetic crosstalk with longer duration on pulse compression results, the electromagnetic crosstalk is removed as shown in Figure 27b, and Figure 27c shows the crack-reflected wave after pulse compression. After the Barker code pulse compression processing, the main lobe width of the crack-reflected wave is reduced, which is beneficial for improving the resolution.

**Figure 28:**
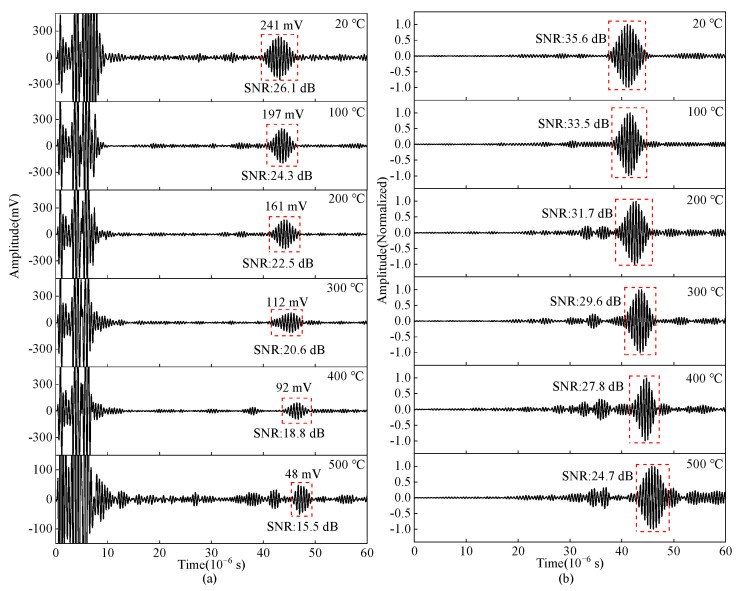
Crack-reflected wave obtained by (**a**) tone-burst excitation method (**b**) Barker code pulse compression technique in carbon steel specimens from 20–500 °C.

**Figure 29:**
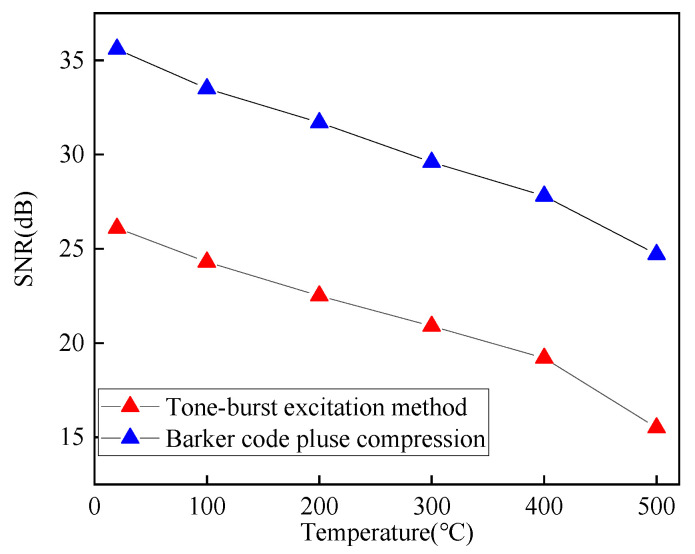
SNR variation curve of the crack-reflected wave with temperatures in carbon steel forgings.

As shown in Figure 28, the ultrasonic signals from 20 °C to 500 °C are obtained by the tone-burst excitation method and the Barker code pulse compression technique, and the SNR variation curve of the crack-reflected wave is shown in Figure 29. Using the tone-burst excitation signal of 2 MHz, the amplitude of the crack-reflected wave is reduced by 80.08%, and the SNR is reduced by 10.6 dB from 20 °C to 500 °C. When the temperature is 500 °C, the SNR of the crack-reflected wave obtained from the tone-burst excitation method is 15.5 dB, while the SNR is improved by 9.2 dB after Barker code pulse compression. Applying the Barker code pulse compression technique to angled SV wave EMAT in high-temperature crack detection can obtain detected waves with a higher SNR.

## 5. Conclusions

The conductivity of the coil copper wire, copper backplate, and steel decrease with increasing temperature, and the equivalent impedance of the MLC increases. The power distribution of the excitation and reception equivalent circuits changes, the excitation current decreases, and the input voltage of the reception circuit decreases, which leads to a reduction of the ultrasonic signal amplitude. For EMAT with Lorentz force as the main conversion mechanism, the radiation angle of the angled SV wave decreases with increasing temperature, the directivity of the acoustic beam is weakened, and the overall change trend of the main lobe amplitude is small. When the temperature increases from 20 °C to 500 °C, the amplitude of the block-corner reflected wave decreases by 64.9%, and the SNR decreases by 11.4 dB.

Impedance-matching methods and impedance-matching parameters significantly impact the pulse compression effect. The pulse compression effect depends mainly on the energy loss due to the matching difference characteristics between the EMAT probe and the electromagnetic ultrasonic detection system. The higher the input voltage of the preamplifier, the larger the defected echo amplitude obtained with the Barker code pulse compression technique. When the temperature is 500 °C, the SNR of the crack-reflected wave received from the Barker code pulse compression technique is improved by 9.2 dB compared to the tone-burst excitation method. Applying the Barker code pulse compression technique to crack detection can improve the SNR of the detected ultrasonic wave signal, effectively solving the problems of poor ultrasonic signal SNR.

The problems of localization and poor SNR of ultrasonic signal in high-temperature forging crack detection by angled SV wave EMAT are addressed. A circuit-field coupled FE model with angled SV wave EMAT in the carbon steel forgings detection process is established based on the Barker code pulse compression technique, and the SNR and resolution are improved after pulse compression through optimized design, which provides theoretical guidance for crack detection in high-temperature metal workpieces.

## Figures and Tables

**Table 1:** Design parameters and values of angled SV wave EMAT.

Parameter	Value/mm	Parameter	Value
magnet width	25	wire width	0.15 mm
magnet height	30	wire spacing	0.3 mm
sample length	160	turns	14
sample height	50	splits	4

**Table 2:** Characteristic parameters of metal materials at different temperatures.

Temperature (°C)	Copper Conductivity (10^7^ S/m)	Steel Conductivity (10^6^ S/m)	Steel Elastic Modulus (GPa)
20	6.00	5.96	212
100	4.60	4.44	210
200	3.49	3.39	206
300	2.80	2.52	199
400	2.32	2.04	190
500	2.00	1.59	180

## Data Availability

The datasets generated from the current study are available from the corresponding author on reasonable request.
